
JOURNAL INFORMATION
==============================
NLM Title Abbreviation: Atlanta J Rec Med
NLM Journal ID: 0001011

Atlanta Journal-Record of Medicine

Publisher: Biomedical Journal Digitization Project

ARTICLE INFORMATION
==============================
PMCID: PMC9038364
Article version: 1
Subjects: Book Reviews

Book Reviews

Print publication date: 1915 Feb
Volume: 61
Issue: 11
First page: 553
Last page: 554
License: This work is free of known copyright restrictions. For more information, please see PMC Back Issue Digitization.
License URL: https://creativecommons.org/publicdomain/mark/1.0/

==============================

**IF YOUR CHOICE OF HYPNOTICS**
**in the presence of a distinct valvular lesion or a myocardial weakness, has been**

**PASADYNE**

(DANIEL'S CONCENTRATED TINCTURE OF PASSIFLORA INCARNATA.)

**you may well know that you have carefully observed the "safety first" rule.**

Although of the highest efficiency in the relief of sleeplessness and nervousness PASADYNE (Daniel) is without immediate or remote evil after-effects.

SAMPLES AND LITERATURE SUPPLIED TO PHYSICIANS PAYING EXPRESS CHARGES.

Laboratory of JOHN B. DANIEL, Atlanta, Ga.

have seen sufferers from the headache of heart disease and edema of the lungs cyanosed from acetanilid (the prominent ingredient of nearly all headache remedies) on account of the cheapness of the drug. The story of the damage and deaths from such treatment is partially told in the numerous sudden deaths reported as heart disease and apoplexy. The Government should force all such preparations to print "Contains acetanilid, a heart depressant and poison," in a prominent place and in large letters on every package and bottle offered for sale to the public.

Hyperacidity of the stomach is a frequent cause of headache. In such cases all food-producing acidity (usually sweets and starches) should be excluded, and lime water, soda or some alkali should be freely administered to neutralize the acidity. This often relieves the headache without the use of other drugs. Many neurotic women, from constipation, lack of nourishment and worry, have what might be called nerve or "brain-storm" headaches. This type often drifts into drug addiction, for which they blame the doctor. Hypodermics of morphine should be used only as a last resort in all such cases. —Medical Council.

### BOOK REVIEWS

A LABORATORY MANUAL AND TEXT-BOOK OF EMBRYOLOGY. By Charles W. Prentiss, A. M., Ph. D., Professor of Microscopic Anatomy in the Northwestern Medical School, Chicago. Octavo of 400 pages, with 368 illustrations, many of them in colors. Philadelphia and London, W. B. Saunders Company, 1915. Cloth, $3.75.

This book combines brief descriptions of vertebrate embryos, which are studied in the laboratory, with an account of human embryology adapted especially to the medical student. There are 368 excellent illustrations.

THE DIAGNOSIS AND TREATMENT OF DIGESTIVE DISEASES.—A practical treatise for students and practitioners of medicine. By George M. Niles, M. D., Professor of Gastroenterology and Clinical Medicine, Atlanta Medical College; Gastroenterologist to the Georgia Baptist Hospital, Wesley Memorial Hospital, Atlanta Hospital; Consulting Gastroenterologist to the Anti-Tuberculosis Association, Atlanta, Georgia. With one colored plate and 86 other illustrations. Philadelphia, P. Blakiston's Sons & Co., 1012 Walnut Street.

In preparing this excellent work, Niles has devoted the main part of his efforts to answer two important questions, namely, "What is the disorder?" and "What should be done for it?

The book is compact, concise, but easily intelligible; it contains descriptions of the various reliable tests for gastric contents, intestinal juices and feces. Clear descriptions are given of the methods of determining the position, size, motility, etc., of the stomach, intestines and other abdominal viscera. A succint statement of the diagnostic methods indicated is also given with an exhaustive discussion of both general and special therapy as applied to these diseases. The work of Niles has been based on actual practice and represents his own experience and thus adds greatly to the value of this book.

---

STUDENT'S MANUEL OF GYNECOLOGY, by John Osburn Polak, M. Sc., M. D., F. A. C. S. Professor of Obstetrics and Gynecology, Long Island College Hospital; Professor of Obstetrics in the Dartmouth Medical School; Gynecologist to the Jewish Hospital; Consulting Gynecologist to the Bushwick, Coney Island, Deaconess', and Williamsburg Hospitals, Brooklyn, and the People's Hospital, New York; Fellow American Gynecological Society, New York Academy of Medicine, etc. Illustrated with 100 engravings and nine plates, Lea & Febiger, Philadelphia.

This splendid little volume is intended primarily for the student and practitioner who desire the essential facts of gynecology briefly and clearly stated. Polak gives the indications for surgical intervention and detailed descriptions of some of the usual gynecologic operations.
